# Connaissances, perceptions et attitudes des auxiliaires et assistants médicaux sur la télémédecine au Togo

**DOI:** 10.48327/mtsi.v5i4.2025.744

**Published:** 2025-09-03

**Authors:** Kokou ADAMBOUNOU, Rarba Baniki MOROUMA-TISSOGA, Akoété Beleave KOUEVIDJIN

**Affiliations:** Unité de télé médecine, Centre hospitalier universitaire (CHU) Campus de Lomé/Laboratoire de biophysique et imagerie médicale, Université de Lomé, Togo

**Keywords:** Télémédecine, Télésanté, Santé numérique, Auxiliaires et assistants médicaux, Togo, Afrique Subsaharienne, Telemedicine, Telehealth, Digital health, Medical auxiliaries and assistants, Togo, Sub-Saharan Africa

## Abstract

Lobjectif de cette étude est d’évaluer les connaissances, perceptions et attitudes des auxiliaires et assistants médicaux sur la télémédecine au Togo.

**Méthodologie:**

Il s’agit d’une étude transversale descriptive effectuée du 27 mars au 26 juin 2021 incluant les auxiliaires et assistants médicaux exerçant au Togo. Les questionnaires ont été renseignés par voie électronique en ligne via Google Forms et physiquement sur format imprimé.

**Résultats:**

Sur les 315 auxiliaires et assistants médicaux interrogés, 76,2% étaient des auxiliaires (dont 81,7% d’infirmiers et sages-femmes) et 23,8% des assistants médicaux. Ils étaient 76,8% à avoir au moins une fois entendu parler de la télémédecine. Ils connaissaient la téléconsultation (53%), la télésurveillance (18,1%) et la téléassistance (15,2%). Les principaux freins au développement de la télémédecine évoqués étaient les difficultés économiques (64,8%) et les freins organisationnels (54,3%). Ils savaient que le consentement du patient avant tout acte de télémédecine est nécessaire dans 48,6% des cas. Ils estimaient pour 82,2% d’entre eux que la télémédecine est utile dans la pratique médicale quotidienne et manifestaient presque tous (90,5%) lenvie de se former à la télémédecine. Ils pratiquaient la télésurveillance (27,4%) et la téléassistance médicale (24,2%). Ils étaient 70,5% à utiliser WhatsApp pour prescrire une ordonnance à distance et 49,8% à surveiller un patient à distance.

**Conclusion:**

Avec un niveau de connaissance peu satisfaisant, les auxiliaires et assistants médicaux au Togo avaient une perception encourageante sur la télémédecine, mais les bonnes attitudes n’étaient pas toujours adoptées.

## Abstract (en)

**Objective:**

This study aims to evaluate the knowledge, perceptions, and attitudes of medical auxiliaries and assistants regarding telemedicine in Togo.

**Methodology:**

This descriptive cross-sectional study was conducted from March 27 to June 26, 2021, and included medical auxiliaries and assistants working in Togo. Participants completed the questionnaires electronically via Google Forms or in printed format.

**Results:**

Of the 315 medical auxiliaries and assistants surveyed, 76.2% were auxiliaries (including 81.7% nurses and midwives), and 23.8% were assistants. Seventy-six point eight percent had heard of telemedicine at least once. They were familiar with teleconsultation (53%), telemonitoring (18.1%), and telehealth assistance (15.2%). The main barriers to developing telemedicine that were cited were economic difficulties (64.8%) and organizational barriers (54.3%). They knew that patient consent is required for telemedicine procedures in 48.6% of cases. Eighty-two percent of them considered telemedicine useful for everyday medical practice, and nearly all (90.5%) wanted training in telemedicine. They practiced telemonitoring (27.4%) and telehealth assistance (24.2%). Seventy-five percent used WhatsApp to prescribe remotely, and 49.8% used it to monitor patients remotely.

**Conclusion:**

Despite their limited knowledge, medical auxiliaries and assistants in Togo had a positive perception of telemedicine; however, they did not always adopt good attitudes.

## Introduction

La télémédecine est une pratique médicale à distance utilisant les technologies de l’information et de la communication (TIC) [8]. La plupart de ces technologies sont audiovisuelles. La télémédecine apporte, à ce titre, une nouvelle vision de la prise en charge médicale. Elle est considérée comme l’une des principales innovations dans le domaine des services de santé, non seulement du point de vue technologique mais aussi culturel et social, car elle facilite l’accès à ces services et améliore la qualité des soins médicaux et l’efficacité organisationnelle [5]. Elle permet de pallier le manque de ressources humaines qualifiées en santé et la distance géographique en s’appuyant sur les TIC, apportant ainsi l’expertise là où elle n’existe pas [21,23]. L’avancée de la technologie et l’accroissement des besoins en santé ont favorisé l’implémentation des solutions de télémédecine sur les cinq continents. Depuis le début de la pandémie de la Covid-19, le recours à la télémédecine pour surmonter certaines difficultés a connu une croissance importante. La télémédecine a permis de suivre les patients souffrant de maladies chroniques lorsqu’ils étaient confinés chez eux, de réduire l’affluence dans les centres de santé pour minimiser le risque de propagation de la maladie [15,16]. Plus que jamais l’importance de la télémédecine est devenue une évidence.

Contrairement à l’Europe où plus de 60% des pays disposent de services de télémédecine, la majorité des initiatives dans ce domaine ne dépasse pas en Afrique le stade de projets pilotes [18]. Au Togo par exemple, les premières initiatives ont véritablement commencé après les années 2010, notamment avec la plateforme « moindre coût » expérimentée entre le CHU Campus, le CHU de Tours (France) et le CHR de Tsévié (Togo) [2]. La durabilité d’un projet de télémédecine dépend en partie de l’adhésion non seulement des médecins mais aussi des autres professionnels de santé qui sont impliqués dans sa conception et sa mise en œuvre. Cette adhésion dépend des connaissances et perceptions que les médecins et les autres soignants (auxiliaires et assistants médicaux) ont de la télémédecine. Malheureusement en Afrique, si quelques études ont été faites sur la connaissance et la perception de la télémédecine par les médecins, rares sont celles qui concernent les auxiliaires et/ou assistants médicaux.

C’est dans ce contexte que nous avons entrepris cette étude qui a pour objectif général d’évaluer les connaissances, perceptions et attitudes des auxiliaires et assistants médicaux sur la télémédecine au Togo.

## Cadre et méthodes d’étude

Il s’est agi d’une étude transversale descriptive effectuée du 27 mars au 26 juin 2021 portant sur les connaissances, perceptions et attitudes des auxiliaires et assistants médicaux vis-à-vis de la télémédecine au Togo. Le Togo est situé en Afrique occidentale, avec une superficie de 56 600 km^2^ et une population estimée à 8,5 millions d’habitants en 2020 [22]. Selon un rapport de l’Autorité de régulation des postes et télécommunications du pays, la proportion de la population connectée à l’Internet fixe et mobile est de 61,7% en 2019, avec un nombre d’abonnés à haut débit fixe en constante augmentation [17]. Six opérateurs sont présents sur le marché des communications électroniques et offrent des services de téléphonie fixe, de téléphonie mobile, d’Internet fixe (technologies FTTH, ADSL et Wimax) et d’Internet mobile (2G, 3G, 4G et 5G). Le taux de pénétration du haut débit fixe et mobile a atteint 44% en 2019 [17].

Le Togo a connu jusqu’à 2021, année de l’étude, des initiatives ou activités de télémédecine dont les plus marquantes sont celles de l’unité de télémédecine du CHU Campus de Lomé (avec le projet *Indian Pan African e-Network,* celui de télé-échographie avec le CHR Tsévie, et celui de la plateforme Telemed CHU Campus), celles de télé-radiologie entre l’hôpital de Blitta et le CHU-Campus, celles encore de télé-éducation médicale du Réseau africain francophone de télémédecine (RAFT) et enfin celles de télédermatologie avec la Société togolaise de dermatologie (SOTODERM). Plusieurs applications de e-santé pilotées par des organisations diverses étaient également recensées en 2021. Il s’agissait notamment de l’application *DokitaEyes* qui est un carnet de santé numérique, l’application de régulation médicale *Yo Dokita,* l’application *InfoAdo Jeunes* qui opère dans le domaine de la santé mobile, la santé sexuelle et reproductive et la planification familiale, les applications *Ecentre convivial* et *Kondjigbale* dans le domaine de la santé mobile, et la web *TV SOS Docteur TV.*

Ont été inclus dans l’étude les auxiliaires et assistants médicaux exerçant au Togo aussi bien en structure publique que privée.

Le Code de santé publique du Togo, dans son article 159, distingue les auxiliaires médicaux comme suit [12]: sage-femme d’État, infirmier(ère) d’État, technicien supérieur en soins infirmiers et obstétricaux, technicien de laboratoire, masseur kinésithérapeute, maître orthoprothésiste, technicien orthoprothésiste d’État, technicien biomédical, technicien orthophoniste, accoucheuse auxiliaire d’État, infirmier(ère) auxiliaire d’État et auxiliaire en pharmacie.

Le métier d’assistant médical, reconnu par le Code de santé publique togolais dans son article 159 comme profession paramédicale, est défini comme une profession de santé que l’on peut exercer sans être titulaire d’un diplôme de doctorat en médecine ou en chirurgie dentaire reconnu par l’État [12]. Il s’agit entre autres des techniciens supérieurs de santé (médecine générale, manipulation en radiologie et imagerie médicale, en instrumentation chirurgicale, en odontostomatologie, en ophtalmologie et en ORL).

Les paramètres de l’enquête concernaient les caractéristiques sociodémographiques, les connaissances, la perception et l’attitude des auxiliaires et assistants médicaux vis-à-vis de la télémédecine. Les connaissances désignent l’ensemble des notions objectives acquises par les enquêtés sur la télémédecine. Les perceptions désignent l’ensemble des points de vue subjectifs des enquêtés sur ce sujet, et les attitudes l’ensemble des comportements ou pratiques adoptés en la matière par les enquêtés.

Ces paramètres ont servi à l’élaboration de notre questionnaire qui comportait des questions à réponses ouvertes et courtes, des questions à choix multiples et des questions fermées.

Nos données ont été collectées par voie électronique et par voie physique. Nous avons enregistré notre formulaire sur Google Forms sur l’adresse *uniform resource locator* (URL) suivante: https://forms.gle/MSgdEvk2TSSC5squ8. Ce lien URL a été envoyé par messagerie (e-mail et WhatsApp) aux auxiliaires et assistants médicaux, principalement *via* les différentes plateformes de groupes WhatsApp des auxiliaires et assistants médicaux disponibles pendant la période d’étude. Les plateformes de groupes WhatsApp ciblées étaient notamment celles créées par les différentes associations des auxiliaires et assistants médicaux du Togo et celles des différents hôpitaux du pays. Nous avons imprimé des fiches physiques que nous avons fait remplir par les prestataires directement sur site en notre présence.

Tous les participants ont été informés de l’objectif de l’étude et ont été invités à y participer s’ils y consentaient. Toutes les informations relatives à l’étude ont été mentionnées dans le corps du texte du formulaire Google. Ils ont également été informés de leur droit de refuser de participer ou de se retirer à tout moment du processus de collecte de l’étude. Le remplissage des fiches par les enquêtés a été strictement anonyme.

Le traitement et l’analyse statistique des données ont été réalisés à l’aide du logiciel EPI INFO version 7. 2. 2. 6. Les tests statistiques du Chi2 ou de Fischer ont été effectués pour les variables quantitatives et nous avons considéré qu’une différence était statistiquement significative si la valeur de p était inférieure à 0,05.

## Résultats

Notre échantillon était constitué de 315 auxiliaires et assistants médicaux dont 175 hommes et 140 femmes soit un sex-ratio de 1,25. Les enquêtés étaient à 76,2% des auxiliaires médicaux, avaient majoritairement moins de 35 ans (51,4%) et un niveau licence (78,7%). Ils exerçaient depuis moins de 10 ans (58,1%) (Tableau I).

**Tableau I:** Répartition des auxiliaires et assistants médicaux en fonction de leurs caractéristiques sociodémographiques au Togo, 2021

	n	%
Âge (en années)	315	
<25	38	12,1
[25-35 [	124	39,4
[35-45 [	114	36,2
[45-55 [	35	11,1
[55-65 [	2	0,6
[65-75]	2	0,6
Qualification	315	
Auxiliaires médicaux	240	76,2
Assistants médicaux	75	23,8
Expérience professionnelle (en années)	315	
< 5 (n=89)	89	28,3
[5-10] (n=94)	94	29,8
[10-15] (n=82)	82	26,0
>15 (n=50)	50	15,9
Niveau d’étude	315	
Certificat d’études du premier degré CEPD	2	0,6
Brevet d’études du premier cycle BEPC	19	6,0
BAC	23	7,3
Licence	248	78,7
Master	23	7,3

Les 240 auxiliaires médicaux comprenaient notamment 114 infirmiers (47,5%) et 82 sagesfemmes (34,2%). Les 75 assistants médicaux étaient majoritairement assistants médicaux de médecine générale (53,3%), encore appelés techniciens supérieurs de santé.

Trois cent huit (97,8%) des enquêtés avaient été formés au Togo. Ils étaient 221 (70,2%) à exercer leurs fonctions à Lomé et 94 (29,8%) dans les autres villes du pays. Deux cent quatre-vingtquatre soit 90,2% d’entre eux exerçaient au sein des structures publiques et le reste (9,8%) dans des structures privées.

La vitesse de la connexion Internet dans leur lieu d’exercice était jugée faible par 87 d’entre eux (27,6%), médiocre par 100 (31,8%), bonne par 77 (24,4%) et très bonne par 8 (2,5%) des enquêtés. La connexion Internet n’était pas accessible dans le lieu d’exercice de 43 enquêtés (13,7%).

Deux cent quarante-deux soit 76,8% des praticiens enquêtés ont déclaré avoir au moins une fois entendu parler de télémédecine. Les médias étaient le moyen d’information le plus cité par les enquêtés (Tableau II).

**Tableau II:** Répartition des moyens d’information des auxiliaires et assistants médicaux ayant déjà entendu parler de la télémédecine selon leurs villes d’exercice au Togo, 2021

	Lomé		Hors de Lomé		Total	
	n	%	n	%	n	%
Confrère	35	14,5	17	7,0	52	21,5
Internet	64	26,4	25	10,3	89	36,8
Patients	6	2,5	0	0	6	2,5
Réunion	12	5,0	7	2,9	19	7,9
Revues médicales	14	5,8	8	3,3	22	9,1
Soirée thématique	3	1,2	1	0,4	4	1,7
Médias	72	29,7	33	13,6	105	43,4

Soixante-six enquêtés (21%) avaient entendu parler d’une activité ou d’un projet de télémédecine au Togo. Sur les 315 enquêtés, 35,9% ne connaissaient aucun acte de télémédecine, 53% connaissaient l’acte de téléconsultation, 18,1% l’acte de télésurveillance médicale, 15,2% l’acte de téléassistance médicale, 8,9% l’acte de téléexpertise et 3,2% l’acte de régulation médicale.

La connaissance de la téléconsultation et de la téléexpertise était davantage retrouvée chez les enquêtés ayant un niveau d’étude universitaire, avec une différence statistiquement significative (p<0,05), tandis que celle de la télésurveillance et de la téléassistance était significativement (p<0,05) plus souvent retrouvée chez les praticiens exerçant à Lomé (Tableau III). Le Tableau III montre également que la connaissance de la téléassistance était davantage exprimée au sein des enquêtés ayant un niveau d’étude universitaire avec une différence statistiquement significative (p<0,05). Soixante-onze enquêtés (soit 22,5%) avaient déclaré connaître des moyens pour apprendre à pratiquer la télémédecine dont 41 (soit 13%) avaient cité la visioconférence et 36 (soit 11,4%) la e-formation. Cent cinquante-trois enquêtés (48,6%) savaient qu’il est important d’avoir le consentement du patient avant le recueil et le partage de ses informations avec d’autres collègues ou avant tout acte de télémédecine. La connaissance de l’importance du consentement du patient avant un acte de télémédecine était plus fréquente chez les assistants médicaux et chez les enquêtés exerçant hors de Lomé (p < 0,05) (Tableau IV).

**Tableau III:** Profil des auxiliaires et assistants médicaux connaissant les différents actes de la télémédecine au Togo, 2021

	Téléconsultation			Téléexpertise			Télésurveillance			Téléassistance		
	n	%	p	n	%	p	n	%	p	n	%	p
Âge (en années)			0,482			0,154			0,170			0,176
< 35 (n=162)	89	54,9		18	11,11		34	21,0		29	17,9	
> 35 (n=153)	78	51,0		10	6,5		23	15,0		19	12,4	
Expérience professionnelle (en années)			0,567			0,077			0,908			0,634
< 5 (n=89)	49	55,0		9	10,1		16	18,0		16	18,0	
[5-10] (n=94)	50	53,2		9	10,0		17	18,1		13	13,8	
[10-15] (n=82)	43	52,4		9	11,0		16	19,5		11	13,4	
>15 (n=50)	25	50,0		1	2,0		8	16,0		8	16,0	
Lieu d’exercice			0,006			0,878			0,004			0,030
Lomé (n=221)	106	48,0		20	9,0		49	22,2		40	18,0	
Hors de Lomé (n=94)	61	64,9		8	8,5		8	8,5		8	8,5	
Niveau d’étude			0,017			0,022			0,094			0,009
Non universitaire (n=44)	16	36,4		0	0		4	9,1		1	2,3	
Universitaire (n=271)	151	55,7		28	10,3		53	19,6		47	17,3	
Qualification			0,165			0,121			0,844			0,344
Auxiliaire (n=240)	122	50,8		18	7,5		44	18,3		34	14,2	
Assistant (n=75)	45	60,0		10	13,3		13	17,3		14	18,7	

**Tableau IV:** Profil des enquêtés ayant cité la e-formation et la visio-conférence comme moyen de formation à la pratique de la télémédecine et ceux connaissant la nécessité d’avoir le consentement du patient avant un acte de télémédecine au Togo, 2021

	e-formation			Visioconférence			Consentement du patient		
	n	%	p	n	%	p	n	%	p
Âge (en années)			0,378			0,977			0,877
< 35 (n=162)	21	13,0		21	13,0		78	48,2	
> 35(n=153)	15	9,8		20	13,1		75	49,0	
Expérience professionnelle (en années)			0,539			0,886			0,760
< 5 (n=89)	8	9,0		5	5,6		43	48,3	
[5-10] (n=94)	14	14,9		19	20,2		45	47,9	
[10-15] (n=82)	13	15,9		16	19,5		39	47,6	
>15 (n=50)	1	2,0		1	2,0		26	52,0	
Lieu d’exercice			0,005			<0,001			<0,001
Lomé (n=221)	18	8,1		18	8,1		91	41,2	
Hors de Lomé (n=94)	18	19,2		23	24,5		62	66,0	
Niveau d’étude			0,599			0,002			0,656
Non universitaire (n=44)	4	9,1		0	0		20	45,5	
Universitaire (n=271)	32	11,8		41	15,1		133	49,1	
Qualification			0,154			0,039			0,045
Auxiliaire (n=240)	24	10,0		26	10,8		109	45,4	
Assistant (n=75)	12	16,0		15	20,0		44	58,7	

Les principaux bénéfices à réaliser des actes de télémédecine cités par les 315 enquêtés étaient les bénéfices pour les patients (44,4%), la lutte contre la désertification médicale dans les zones rurales (19,7%) et le rapprochement avec une structure hospitalière (19,1%).

Les principaux freins de la télémédecine cités par les enquêtés étaient: les freins économiques (64,8%), les freins organisationnels (54,3%), le manque de motivation (37,1%), le manque de crédibilité par rapport aux patients (36,8%) et l’examen incomplet (35,6%).

Deux cent cinquante-neuf enquêtés (82,2%) jugeaient utile la télémédecine dans leurs pratiques quotidiennes. La télémédecine était perçue comme une nécessité par 142 enquêtés (45,1%), comme l’espoir et l’avenir de la médecine par 133 (42,2%) et une affaire de médecin spécialiste par 26 (8,3%). Ils étaient 285 enquêtés (90,5%) à avoir manifesté une envie de se former à la télémédecine. Les praticiens enquêtés exerçant hors de Lomé percevaient davantage la télémédecine comme une nécessité avec une différence statistiquement significative (p<0. 05); ceux exerçant à Lomé et les auxiliaires médicaux la percevaient plus comme un espoir et un avenir de la médecine avec une différence statistiquement significative (p<0. 05) (Tableau V). Le Tableau V montre également que l’envie de se former était significativement (p<0. 05) plus exprimée par les praticiens de plus de 35 ans, ceux ayant plus de 15 ans d’expérience professionnelle et ceux exerçant hors de Lomé. La médecine générale était le premier domaine devant bénéficier du développement de la télémédecine (74,3%) selon les enquêtes, suivie respectivement par l’imagerie médicale (41%), la dermatologie (38,4%), la pédiatrie (29,2%), la chirurgie (27%), la cardiologie (26,7%), la psychiatrie (26,7%) et la gynéco-obstétrique (6,4%). Parmi les enquêtés, 27,3% ont déclaré pratiquer l’acte de télésurveillance, 24,1% la téléassistance médicale, 9,2% l’acte de téléexpertise et 3,5% la régulation médicale. Cent soixante-neuf des enquêtés (53,7%) avaient déclaré n’avoir jamais pratiqué aucun acte de télémédecine. La pratique de la télésurveillance médicale était significativement (p<0,05) plus retrouvée chez les plus jeunes (moins de 35 ans), et chez ceux ayant une expérience professionnelle de moins de 5 ans (Tableau VI).

**Tableau V:** Profil des enquêtés considérant la télémédecine comme une nécessité, un espoir et un avenir de la médecine, et ceux ayant envie de se former à la pratique de la télémédecine au Togo, 2021

	Télémédecine comme nécessité			Télémédecine comme espoir et avenir de la médecine			Envie de se faire former à la télémédecine		
	n	%	p	n	%	p	n	%	p
Âge (en années)			0,361			0,050			0,032
< 35 (n=162)	69	42,6		77	47,5		141	87,0	
> 35(n=153)	73	47,7		56	36,6		14	94,1	
Expérience professionnelle (en années)			0,326			0,367			0,046
< 5 (n=89)	36	40,5		40	44,9		78	87,6	
[5-10] (n=94)	43	45,7		42	44,7		82	87,2	
[10-15] (n=82)	39	47,6		31	37,8		77	94,0	
>15 (n=50)	24	48,0		20	40,0		48	96,0	
Lieu d’exercice			0,004			0,016			0,038
Lomé (n=221)	88	39,8		103	46,6		195	88,2	
Hors de Lomé (n=94)	54	57,5		30	31,9		90	96,0	
Niveau d’étude			1,0			0,849			1,000
Non universitaire (n=44)	20	45,5		18	41,0		40	90,9	
Universitaire (n=271)	122	45,0		115	42,4		245	90,4	
Qualification			0,1			0,0			0,3
Auxiliaire (n=240)	102	42,5		109	45,4		215	89,6	
Assistant (n=75)	40	53,3		24	32,0		70	93,3	

**Tableau VI:** Profil des auxiliaires et assistants médicaux ayant déjà pratiqué la télésurveillance et la téléassistance médicale au Togo en 2021

	Pratique de la télésurveillance médicale			Pratique de la téléassistance médicale		
	n	%	p	n	%	p
Âge (en années)			0,045			0,592
< 35 (n=162)	52	32,1		41	25,3	
> 35(n=153)	34	22,2		35	22,9	
Expérience professionnelle (en années)			0,043			0,481
< 5 (n=89)	30	33,7		19	21,4	
[5-10] (n=94)	26	27,7		24	25,5	
[10-15] (n=82)	21	25,6		19	23,2	
>15 (n=50)	9	18,0		14	28,0	
Lieu d’exercice			0,328			0,314
Lomé (n=221)	57	25,8		50	22,6	
Hors de Lomé (n=94)	29	30,9		26	27,7	
Niveau d’étude			0,3			0,6
Non universitaire (n=44)	9	20,5		9	20,5	
Universitaire (n=271)	77	28,4		67	24,7	
Qualification			0,1			0,6
Auxiliaire (n=240)	71	29,6		56	23,3	
Assistant (n=75)	15	20,0		20	26,7	

Parmi les personnels enquêtés, 155 (soit 49,2%) et 88 (soit 27,9%) n’avaient jamais utilisé de moyens de consultation et de prescription médicale à distance. Quatre-vingt-sept d’entre eux (27,6%) et 105 (33,3%) n’avaient respectivement jamais utilisé de voies virtuelles de réception de résultats paracliniques de la part des patients et de la part des collègues. L’application WhatsApp était le principal outil non seulement de surveillance et de prescription médicale à distance, mais aussi de réception de résultats de la part des patients et des collègues, utilisé par les enquêtés (Tableau VII). Deux cent quatre-vingt-seize soit 94,0% des enquêtés avaient déclaré n’avoir jamais utilisé d’objets connectés pour la prise de constantes des patients contre 19 (soit 6%) qui ont déclaré avoir déjà utilisé un objet connecté.

**Tableau VII:** Répartition des moyens de surveillance, de prescription à distance et des voies virtuelles de réception des résultats paracliniques utilisés par les auxiliaires et assistants médicaux

	Surveillance à distance		Prescription à distance		Réception de résultats de la part des patients		Réception de résultats de la part des collègues	
	n	%	n	%	n	%	n	%
WhatsApp	157	49,8	222	70,5	222	70,5	206	65,4
Facebook	4	1,3	5	1,6	1	0,3	1	0,3
Autres réseaux sociaux	18	5,7	15	4,8	15	4,8	3	1,0

Les lieux d’exercice de la télémédecine préférés des 315 assistants et auxiliaires médicaux étaient les structures publiques (65,4%), leur domicile (50,5%) et les cabinets libéraux (43,8%).

## Discussion

En incluant les auxiliaires médicaux et les assistants médicaux, notre étude se veut être une étude consacrée aux soignants non médecins dont le rôle dans la prise en charge des patients est incontournable dans toute structure sanitaire. Les auxiliaires médicaux étaient majoritairement (81,8%) des infirmiers et des sages-femmes dans notre étude, professions les plus nombreuses et les plus impliquées aux côtés des médecins dans cette prise en charge en milieu hospitalier.

Au Togo, la pénurie de médecins notamment dans les zones rurales a amené les autorités sanitaires du pays à former des professionnels de santé intermédiaires entre les auxiliaires médicaux et les médecins généralistes. Ils assurent les fonctions de médecin généraliste par délégation dans les centres de santé ne disposant pas de médecin. Les assistants médicaux de médecine générale, encore appelés techniciens supérieurs de santé conformément au Code de la santé du Togo [12], sont les plus opérationnels et donc logiquement majoritaires (53,3%) dans notre échantillon.

Le sex-ratio des enquêtés qui était de 1,25 est différent de celui retrouvé par Ayatollahi *et al.* (0,87) dans l’étude sur les connaissances et perceptions des cliniciens sur la télémédecine en Iran [4]. Notre résultat est cohérent avec le dernier rapport d’activité du ministère de la Santé et de l’hygiène publique togolais qui retrouvait un sex-ratio de 1,30 au sein du personnel de santé dans le secteur public [14]. Il traduit la représentativité de notre échantillon en termes de genre.

On peut toutefois regretter qu’environ le quart des praticiens enquêtés dans notre étude n’ait jamais entendu parler de la télémédecine en 2021 alors que la pandémie de la Covid-19 a médiatisé l’utilité de la télémédecine qui s’est révélée être une des solutions de mise en œuvre de la distanciation sociale pendant cette période. Ce résultat peut donc traduire une faible communication sur la télémédecine par les autorités sanitaires togolaises lors de la pandémie apparue en 2019.

Comparativement à notre étude où les médias étaient le moyen d’information sur la télémédecine le plus cité par les enquêtés (43%), suivi par l’Internet (36,8%) et les confrères des enquêtés (21,5%), Shittu *et al.,* dans leur étude au Nigeria, ont aussi trouvé que les moyens d’information majoritairement cités sur la télémédecine étaient les mêmes: les médias, suivis d’Internet et des confrères [19]. Dans leur enquête portant sur la télémédecine parmi les médecins de Milan en Italie en 2005, Gaggioli *et al.* avaient trouvé les revues médicales en première place (46%), suivies par les collègues (41%), les conférences scientifiques (26%), la télévision (19%) et l’Internet (10%) [10]. Dans son enquête auprès des médecins généralistes de Gironde, France, en 2018, Messon trouvait en première position les revues médicales (66%), suivies par Internet (52,1%), les confrères (48,3%), la télévision (42,3%) et les patients (6%) [13]. La première place occupée par les revues médicales dans les études réalisées en Occident (Italie et France), contrairement aux études africaines (la nôtre et celle du Nigeria), peut s’expliquer par une plus grande accessibilité des revues médicales pour les médecins dans ces pays. La télémédecine se décline en cinq principaux actes: la téléconsultation, la téléexpertise, la télésurveillance, la téléassistance médicale et la régulation médicale. Toutefois, 36% environ des enquêtés de notre étude ne connaissaient aucun acte de télémédecine. La téléconsultation, connue par 53% des enquêtés, permet à un professionnel médical de donner une consultation à distance à un patient. La téléexpertise, méconnue par environ 81% des enquêtés, permet à un professionnel médical de solliciter à distance l’avis d’un ou de plusieurs professionnels médicaux en raison de leur formation ou de leurs compétences particulières, sur la base des informations médicales liées à la prise en charge d’un patient. Le patient n’assiste pas à la séance. La télésurveillance médicale a pour objet de permettre à un professionnel médical d’interpréter à distance les données nécessaires au suivi médical d’un patient et, le cas échéant, de prendre des décisions relatives à sa prise en charge. L’enregistrement et la transmission des données peuvent être automatisés ou réalisés par le patient lui-même ou par un professionnel de santé. Elle était connue par seulement 18. 1% des enquêtés, alors qu’elle implique souvent les auxiliaires médicaux comme les infirmiers et les sages-femmes. La téléassistance médicale, connue par 15,2% des enquêtés, permet à un professionnel médical d’assister à distance un autre professionnel de santé au cours de la réalisation d’un acte médical. Enfin, la régulation médicale (l’acte le moins connu des enquêtés, seulement 3. 1%) est un acte médical pratiqué au téléphone (ou au moyen de tout autre dispositif de télécommunication) par un médecin régulateur. Il a pour but de déterminer et de déclencher dans les meilleurs délais la réponse médicale adaptée à chaque situation. La téléassistance et la télésurveillance médicales étaient plus connues par les praticiens exerçant à Lomé. Ce résultat peut s’expliquer par le fait que les grandes villes comme Lomé (capitale togolaise) offrent plus d’opportunités d’informations sur les avancées médicales que les zones rurales où l’accès à l’information est souvent limité. Dans une enquête réalisée auprès des médecins généralistes exerçant dans les Maisons de santé pluri-professionnelles en Bourgogne, France, en 2019, Berou trouvait 82,9% de médecins qui connaissaient l’acte de téléconsultation, 80% l’acte de téléexpertise, 47,7% la télésurveillance médicale et 20% la téléassistance médicale [6]. Les résultats de Berou, logiquement plus satisfaisants que les nôtres, s’expliquent entre autres par le fait que son étude concernait les médecins exerçant dans un pays développé.

Les actes de télémédecine doivent être encadrés par les exigences déontologiques de la pratique de la médecine dans lesquelles figure en bonne place le consentement du patient [7,20]. Toutefois, seulement 48,6% des praticiens enquêtés savaient qu’il est important d’obtenir ce consentement avant un acte de télémédecine.

Nos résultats montrent que les causes économiques ont été plus fréquemment citées au Togo comme frein à la télémédecine qu’en Gironde. Messon avait retrouvé 55% de médecins évoquant notamment le frein économique du financement dans son étude [13] *versus* 65% dans la nôtre. Ce constat reflète la réalité des pays en développement où les freins économiques et organisationnels restent une barrière majeure au développement de la télémédecine. En effet, la plupart des initiatives de télémédecine en Afrique subsaharienne ne dépassent souvent pas le stade de projet pilote. Les freins économiques sont principalement liés au coût élevé des équipements, à l’accès limité à l’Internet haut débit, au faible budget des structures de santé et à l’absence de modèle économique viable. Les freins organisationnels de la télémédecine en Afrique sont entre autres constitués par l’absence de protocoles standardisés, le manque de personnel formé, l’intégration difficile aux routines cliniques et les failles dans la gestion des données. Outre les difficultés économiques et organisationnelles, le manque de motivation (souvent lié à la résistance au changement) était un frein évoqué par 37,1% des enquêtés. Un résultat qui pourrait être la conséquence du manque de volonté et d’initiative ou la peur du changement dans nos sociétés, notamment africaines.

La télémédecine dans la pratique médicale quotidienne était jugée utile par 82,2% des enquêtés. Shittu *et al.* trouvaient au Nigeria 78,1% des agents de santé indiquant leur volonté d’adopter la télémédecine en prestation de soins si elle était disponible [19]. De même, Ayatollahi *et al.* en 2013, dans leur étude en Iran, rapportaient que 74,8% des cliniciens étaient d’accord avec la nécessité d’utiliser les technologies de télémédecine [4]. Par contre, en France, Messon retrouvait seulement 46% de médecins en Gironde qui trouvaient utile la télémédecine dans la pratique médicale quotidienne [13]. Cette meilleure perception de la télémédecine dans les pays en développement, notamment au Togo, est encourageante et constitue donc une opportunité d’essor de la télémédecine dans ce pays. Les enquêtés exerçant hors de Lomé percevaient plus la télémédecine comme une nécessité que ceux de Lomé. Ce résultat peut s’expliquer par l’isolement technique et professionnel du personnel de santé exerçant dans les zones rurales, notamment dans nos pays en développement. Ceux-ci sont davantage confrontés au besoin de recourir à des avis de collègues ou de spécialistes exerçant dans la capitale ou dans les grandes villes.

Comme dans notre étude où les applications WhatsApp et Facebook étaient les principaux moyens d’exercice de la télémédecine à distance utilisés par les enquêtés, Albarak *et al.* avaient trouvé qu’à Riyad, Arabie saoudite, environ 72% de médecins interagissaient en 2019 avec leurs patients par courriel ou via les réseaux [3]. Il n’est pas étonnant de trouver les réseaux sociaux tels que WhatsApp et Facebook parmi les principaux moyens connus d’exercice de la télémédecine. Les réseaux sociaux ont connu une forte croissance de leur utilisation depuis leur création (WhatsApp en 2009 et Facebook en 2004), et sont devenus incontournables dans tous les domaines, surtout quand il s’agit de communication. Cependant, certains réseaux sociaux, comme WhatsApp et surtout Facebook, ne sont pas indiqués pour une télémédecine sécurisée. En raison des multiples risques sécuritaires, notamment de confidentialité, qu’ils peuvent faire courir aux patients et aux soignants, ils doivent être évités comme canal de communication virtuelle des données des patients lors des actes de télémédecine. L’une des exigences médico-légales, qu’aussi bien les soignants que les patients ne doivent pas perdre de vue lors d’un acte de télémédecine, est la sécurisation des données transmises. Il est important que, pour l’échange d’informations, une messagerie de santé sécurisée soit utilisée et que le recours aux messageries électroniques personnelles soit exclu.

Nos résultats selon lesquels 46,2% des enquêtés déclarent avoir déjà pratiqué un acte de télémédecine, avec une différence significative pour la télésurveillance chez les plus jeunes (moins de 35 ans), et chez ceux ayant une expérience professionnelle de moins de 5 ans, suggèrent que la pratique de la télémédecine par les auxiliaires et assistants médicaux s’amplifiera dans les années à venir au Togo. Des dispositions doivent être prises pour former ces personnels aux bonnes pratiques de la télémédecine. Des formations continues sur la télémédecine, indispensables à tout professionnel de santé désirant intégrer la télémédecine dans sa pratique quotidienne [9], sont d’ailleurs vivement sollicitées par la grande majorité (90,5%) des enquêtés de notre étude. Les objets connectés sont de plus en plus utilisés dans les pays occidentaux pour la surveillance des patients notamment à distance. En France par exemple, le tiers de soignants y avait recours pour suivre les patients selon une étude publiée par Le Guillou *et al.* [11]. Tel n’est pas encore le cas au Togo, en particulier chez les auxiliaires et assistants médicaux qui étaient seulement 6% à déclarer avoir déjà utilisé un objet connecté pour prendre ou surveiller à distance les constantes d’un patient.

Enfin, la majorité des enquêtés ont jugé que la vitesse de la connexion Internet dans leur lieu d’exercice n’était pas satisfaisante. C’était également le cas chez les médecins spécialistes de l’imagerie en Afrique subsaharienne francophone dans une étude antérieure [1]. Ce résultat montre que l’accessibilité de la connexion Internet avec une bande passante de qualité et suffisante dans les hôpitaux reste un défi à relever par les autorités sanitaires de nos pays.

L’utilisation de deux voies de collecte des données (électronique et fiche physique imprimée) peut constituer une certaine limite à notre travail qu’il convient de relever. Le remplissage des fiches physiques directement par les enquêtés pourrait biaiser les réponses. De même, certains enquêtés ont pu consulter des documents avant de répondre lors du remplissage électronique des fiches, induisant des biais dans les données.

## Conclusion

Cette étude transversale incluant les soignants non médecins a révélé au Togo un niveau de connaissance des enquêtés relativement peu satisfaisant sur la télémédecine. Leur perception de la télémédecine était cependant encourageante avec une majorité d’entre eux qui la jugeait utile dans leurs pratiques médicales quotidiennes. Les actes les plus pratiqués par ces auxiliaires et assistants médicaux étaient la télésurveillance et la téléassistance médicale. Les canaux virtuels de faible niveau de sécurité de données télétransmises comme WhatsApp étaient les plus utilisés. Des formations médicales continues sur la télémédecine sont souhaitables pour améliorer le niveau de connaissance des auxiliaires et assistants médicaux du Togo, afin de garantir une télémédecine efficiente et sécurisée au profit des patients.

## Approbation éthique et consentement à la participation

Une approbation du comité de thèse de la Faculté des Sciences de la santé de l’université de Lomé a été obtenue. L’étude n’a pas impliqué l’utilisation d’animaux et a été réalisée conformément à la Déclaration d’Helsinki. Le manuscrit n’a été soumis à aucune autre revue (ou site) en partie ou en totalité pour considération. Il est soumis uniquement à ce journal. Les auxiliaires médicaux et les assistants médicaux inclus dans cette étude ont donné leur consentement éclairé pour participer à cette recherche.

## Disponibilité des données et du matériel

Les données utilisées et/ou analysées au cours de la présente étude sont disponibles auprès de l’auteur correspondant sur demande raisonnable.

## Sources de financement

Cette recherche n’a bénéficié d’aucune subvention spécifique de la part d’organismes de financement des secteurs public, commercial ou à but non lucratif.

## Contributions des auteurs

Kokou ADAMBOUNOU: auteur principal, initiateur du travail, recueil des données, méthodologie, révision critique, approbation finale de la version à publier.

Rarba Baniki MOROUMA-TISSOGA: recueil des données, rédaction du manuscrit, approbation finale de la version à publier.

Akoeté Beleave KOUEVIDJIN: contribution à la rédaction du manuscrit, approbation finale de la version à publier.

## Conflit d’intérêt

Les auteurs ne déclarent aucun conflit d’intérêts.

## Annexe 1: Fiche d’enquête

**A- Caractéristiques sociodémographiques des auxiliaires et assistants médicaux**

1 - Sexe: Masculin /___________/ Féminin /___________/

2 - Âge:

< 25 ans /___________/ 26-35 ans /___________/ 36-45 ans /___________/ 46-55 ans /___________/ 56-65 ans/___________/ > 65 ans /___________/

3 - Qualification de l’auxiliaire/assistant médical:

Infirmier diplômé d’état/___________/ Sage-femme/___________/ Kinésithérapeute /___________/ Orthophoniste /___________/ Technicien supérieur de santé (médecine générale) /___________/ Assistant médical/Cadre administratif des soins de santé /___________/ Auxiliaire en anesthésie-réanimation /___________/ Assistant médical en anesthésie-réanimation /___________/ Assistant médical en radiologie et imagerie médicale /___________/ Assistant médical en instrumentation chirurgicale des blocs opératoires /___________/ Assistant médical en odontostomatologie /___________/ Assistant médical en santé mentale /___________/ Autre………………………..

4 - Niveau d’étude: CEPD /___________/ BEPC /___________/ BAC /___________/ Licence /___________/ Master /___________/

5 - Expérience professionnelle (années): 0-4 /___________/ 5-9 /___________/ 10-15 /___________/ > 15 /___________/

6 - Pays de formation: Togo /___________/ Autre………………………..

7 - Lieu d’exercice: Lomé /___________/ Hors de Lomé: ………………………..

8 - Structure administrative d’exercice:

CHU /___________/ CHR /___________/ CHP /___________/ CMS /___________/ Clinique privée /___________/ Autre………………………..

9 - Quelle est la vitesse de la connexion internet dans votre lieu de profession?

Très rapide /___________/ Bonne /___________/ Médiocre /___________/ Pas de connexion internet /___________/

**B - Connaissances des auxiliaires et assistants médicaux à propos la télémédecine au Togo**

10 - Avez-vous déjà entendu parler de télémédecine? Non /___________/ Oui /___________/

11 - Si oui, par quel biais?

Médias /___________/ Revue médicale /___________/ Soirée thématique /___________/ Réunion /___________/ Auprès d’un confrère /___________/ Patients /___________/ Internet /___________/ Autre………………………..

12 - Parmi les actes de télémédecine suivants, le(s)quel(s) connaissez-vous?

Téléconsultation /___________/ Télé expertise /___________/ Télésurveillance médicale /___________/ Téléassistance médicale /___________/ Régulation médicale /___________/ Aucun /___________/

13a - Quelles sont les solutions / applications / moyens d’exercer à distance que vous connaissez?

…………………………………………………………………………………………………………..

…………………………………………………………………………………………………………..

13b - Auxquelles avez-vous recours?

…………………………………………………………………………………………………………..

…………………………………………………………………………………………………………..

14a - Connaissez-vous des moyens de formation pour apprendre à pratiquer la télémédecine? Non /___________/ Oui /___________/

14b - Si oui: le(s)quel(s)?

E-learning /___________/ Visioconférence /___________/ Ddiplôme interuniversitaire (DIU) /___________/ Journée de la télémédecine /___________/

Autre ………………………..

15- Avez-vous déjà entendu parler d’une activité ou projet de télémédecine ancien ou en cours au Togo?

Non /___________/ Oui /___________/

16 - Pour vous quel sera le principal bénéfice dans votre pratique à réaliser des actes de la Télémédecine?

Rapprochement avec une structure hospitalière (contact avec des spécialistes) /___________/

Bénéfices pour les patients (gain de temps/ moins de déplacement) /___________/

Permettre de lutter contre la désertification médicale dans les zones rurales /___________/ Téléformation /___________/

Télésurveillance médicale /___________/ Autre ………………

17 - Quel(s) est (sont) le (les) principal (aux) frein(s) selon vous, pour pouvoir utiliser la Télémédecine?

Frein organisationnel (restructuration pour mettre en place une telle technique) /___________/ Frein économique (financement du développement, rémunération encore floue) /___________/ Manque de motivation /___________/ Manque de temps (peur de perdre du temps avec une telle pratique) /___________/ Manque de crédibilité par rapport aux patients /___________/ Examen incomplet /___________/ Aspect médicolégal /___________/ Aucun /___________/ Autre………………………..

**C- Perceptions des auxiliaires et assistants médicaux en rapport avec la télémédecine au Togo**

18 - Pensez-vous que la télémédecine est utile dans votre pratique quotidienne?

Non /___________/ Oui /___________/

19 - Pour vous la télémédecine c’est:

L’avenir de la médecine /___________/ De la science-fiction /___________/ Une nécessité /___________/ Une affaire de spécialistes /___________/ Un espoir /___________/ Non concerné /___________/

20 - Pour vous dans quels domaines serait-il intéressant de développer des actes de télémédecine?

Médecine générale /___________/ Imagerie médicale /___________/ Dermatologie /___________/ Pédiatrie /___________/ Cardiologie /___________/ Chirurgie /___________/ Psychiatrie /___________/ Autre………………………..

21 - Souhaiteriez-vous vous former à cette pratique? Non /___________/ Oui /___________/

**D - Attitudes des auxiliaires et assistants médicaux en rapport avec la télémédecine au Togo**

22 - Parmi les actes de télémédecine suivants, le(s)quel(s) pratiquez-vous?

Télé expertise /___________/ Télésurveillance médicale /___________/ Téléassistance médicale /___________/ Régulation médicale /___________/

Aucun /___________/

23 - Avez-vous déjà surveillé un(e) patient(e) par l’une de ces voies virtuelles?

WhatsApp /___________/ Facebook /___________/ Autres réseaux sociaux /___________/ Jamais /___________/ Autre………………………..

24 - Avez-vous déjà prescrit une ordonnance à un(e) patient(e) par une ou plusieurs de ces voies virtuelles?

WhatsApp /___________/ Facebook /___________/ Autres réseaux sociaux /___________/ Jamais /___________/ Autre………………………..

25 - Avez-vous déjà reçu de la part d’un(e) patient(e) un résultat paraclinique par une ou plusieurs de ces voies

virtuelles?

WhatsApp /___________/ Facebook /___________/ Autres réseaux sociaux /___________/ Jamais /___________/ Autre………………………..

26 - Avez-vous déjà reçu de la part d’un collègue, un résultat paraclinique par une ou plusieurs de ces voies virtuelles?

WhatsApp /___________/ Facebook /___________/ Autres réseaux sociaux /___________/ Jamais /___________/ Autre………………………..

27 - Avez-vous déjà pris des constantes de patient par un ou plusieurs de ces objets connectés?

Montre intelligente /___________/ Bracelet intelligent /___________/ Balance connectée /___________/ Jamais /___________/

28 - Dans quel(s) lieu(x) seriez-vous d’accord pour pratiquer des actes de télémédecine?

- Votre maison /___________/ Cabinet libéral /___________/ Structure publique /___________/ Aucun /___________/ Autre………………………..

29 - Le consentement du patient est-il acquis pour le recueil et partage de ses informations avec d’autres collègues? *

Non /___________/ Oui /___________/

## References

[1] Adambounou K, Degan A, Kambire F (2023). État des lieux des connaissances des médecins radiologues et nucléaires sur la télé-imagerie médicale en Afrique subsaharienne francophone en 2022. Médecine Nucl.

[2] Adambounou K, Farin F, Boucher A, Adjenou KV, Gbeassor M, N’Dakena K, Vincent N, Arbeille P (2012). Plateforme de télé-imagerie gynéco-obstétricale à « bas prix ». Imagerie de la Femme.

[3] Albarrak AI, Mohammed R, Almarshoud N, Almujalli L, Aljaeed R, Altuwaijiri S, Albohairy T (2021). Assessment of physician’s knowledge, perception and willingness of telemedicine in Riyadh region, Saudi Arabia. J Infect Public Health.

[4] Ayatollahi H, Sarabi FZ, Langarizadeh M (2015). Clinicians’ Knowledge and Perception of Telemedicine Technology. Perspect Health Inf Manag.

[5] Baudino F (2017). La télémédecine doit prendre toute sa place. Constructif.

[7] Bouquier JJ Du droit au consentement au droit et au refus de soins. Rapport adopté lors de la session du Conseil national de l’ordre des médecins des 29 et 30 janvier 2004 (France)..

[8] Durupt M, Bouchy O, Christophe S, Kivits J, Boivin JM (2016). La télémédecine en zones rurales: représentations et expériences de médecins généralistes. Santé publique.

[9] Firn S, Galland J, Rousseau H, Andres E, Salles N, Disdier P, Azzi J, Baumann C, de Korwin JD (2021). La pratique de la télémédecine par les médecins internistes français en 2019. Rev Med Interne.

[10] Gaggioli A, Carlo S, Mantovani F, Castelnuovo G, Riva G (2005). A telemedicine survey among Milan doctors. J Telemed Telecare.

[11] Guillou F, Schuller MP, Benezet O, Stach B, Lofaso P, Zanetti C (2021). Enquête pneumologie libérale et télémédecine avant COVID. Rev. Mal. Respir. Actual January.

[13] Messon T (2017). Quelle est la place des médecins généralistes dans le développement de la télémédecine? Enquête auprès des médecins généralistes de Gironde. Université de Bordeaux;.

[15] Monaghesh E, Hajizadeh A (2020). The role of telehealth during COVID-19 outbreak: a systematic review based on current evidence. BMC Public Health.

[16] Vendeuvre L, Lecygne C, Jeannot JG, Spahni S, Mazouri-Karker S (2020). Télémédecine à l’ère du COVID-19: une révolution? Expérience des hôpitaux universitaires de Genève. Rev Med Suisse.

[18] Ryu S (2012). Telemedicine: opportunities and developments in member states: report on the second Global survey on eHealth 2009 (Global Observatory for eHealth Series, Volume 2). Healthcare Informatics Research.

[19] Shittu L, Adesanya O, Izegbu M, Oyewopo A, Ade A, Ashiru OA (2007). Knowledge and perception of health workers towards tele-medicine application in a new teaching hospital in Lagos. Scientific Research and Essay.

[20] Breslau J, Barr RM, Bohl M, Hoffman T, Boland GW, Sherry C, Kim W, Shah SS, Tilkin M (2013). ACR white paper on teleradiology practice: a report from the Task Force on Teleradiology Practice. J Am Coll Radiol.

[21] Sood S, Mbarika V, Jugoo S, Dookhy R, Doarn CR, Prakash N, Merrell RC (2007). What is telemedicine? A collection of 104 peer-reviewed perspectives and theoretical underpinnings. Telemed J E Health.

[23] Wootton R, Bonnardot L (2010). In what circumstances is telemedicine appropriate in the developing world?. JRSM Short Rep.

