# Supplementary material for: Knowledge, perceptions and attitudes of medical auxiliaries and assistants on telemedicine in Togo
Source: Med Trop Sante Int. 2025 Sep 3;5(4):mtsi.v5i4.2025.744. [Article in French] doi: 10.48327/mtsi.v5i4.2025.744 (PMC12948359; doi:10.48327/mtsi.v5i4.2025.744)
Supplement: Related texts

# Research article: Knowledge, perceptions and attitudes of medical auxiliaries and assistants on telemedicine in Togo

## Introduction

Telemedicine is the practice of medicine from a distance using information and communication technologies (ICT) [8]. Most of these technologies are audiovisual. As such, telemedicine offers a new vision of medical care. Considered one of the main innovations in healthcare services, telemedicine facilitates access to these services and improves the quality of medical care and organizational efficiency, not only from a technological point of view, but also from cultural and social perspectives [5]. Telemedicine helps overcome the lack of qualified healthcare personnel and geographical distances by relying on ICT, thus bringing expertise to areas where it is lacking [21,23]. Technological advances and growing healthcare needs have promoted the implementation of telemedicine solutions across the five continents. Since the start of the pandemic of Covid-19, telemedicine has grown significantly in use to overcome certain difficulties. Telemedicine enables the monitoring of patients with chronic diseases at home and reduces congestion in health centers, minimizing the risk of disease spread [15,16]. The importance of telemedicine has never been of a clearest evidence.

However, unlike in Europe, where more than 60% of countries have telemedicine services, most initiatives in Africa have not progressed beyond the pilot stage [18]. In Togo, for instance, the first initiatives began after 2010, particularly with the “low-cost” platform tested between the CHU Campus, the CHU of Tours (France), and the CHR of Tsevie (Togo) [2]. The sustainability of a telemedicine project depends on the support of not only doctors, but also other healthcare professionals involved in its design and implementation. The adoption of telemedicine depends on the knowledge and perceptions that doctors and other healthcare workers, including auxiliary and medical assistants, have of it. While a few studies have examined doctors’ knowledge and perceptions of telemedicine in Africa, rare are those who have focused on medical auxiliaries and assistants.

In this context, we undertook a study to evaluate medical auxiliaries’ and assistants’ knowledge, perceptions, and attitudes toward telemedicine in Togo.

## Study framework and methods

This descriptive, cross-sectional study focused on the knowledge, perceptions, and attitudes of medical auxiliaries and assistants toward telemedicine in Togo. It was conducted from March 27 to June 26, 2021. Togo is located in West Africa and covers an area of 56,600 km^2^. Its estimated population was 8.5 million in 2020 [22]. According to a report by the Postal and Telecommunications Regulatory Authority of Togo, 61.7% of the population was connected to fixed and mobile internet in 2019, and the number of fixed broadband subscribers has steadily increased [17]. There are six operators in the electronic communications market that offer fixed telephony, mobile telephony, fixed internet (FTTH, ADSL, and WiMAX technologies), and mobile internet (2G, 3G, 4G, and 5G) services. The fixed and mobile broadband penetration rate reached 44% in 2019 [17].

By the time of the study in 2021, Togo has seen a number of telemedicine initiatives and activities until 2021, the most notable of which are the telemedicine unit at the Lomé University Hospital Campus (with the Indian Pan-African e-Network Project, the teleechography project with the Tsévié Regional Hospital Center, the Telemed CHU Campus platform), teleradiology between Blitta Hospital and the University Hospital Campus, telemedicine education by the Francophone African Telemedicine Network (RAFT), and teledermatology with the Togolese Dermatology Society (SOTODERM). Several e-health applications piloted by various organizations were also identified in 2021. These included the DokitaEyes digital health record application; the Yo Dokita medical regulation application; the InfoAdo Jeunes application, which operates in the fields of mobile health, sexual and reproductive health, and family planning; the Ecentre convivial and Kondjigbale mobile health applications; and the SOS Docteur TV web TV application.

The study included medical auxiliaries and assistants working in both public and private settings in Togo.

According to Article 159 of Togo’s Public Health Code, medical auxiliaries are defined as follows [12]: state midwife, state nurse, senior nursing and obstetrics technician, laboratory technician, physiotherapist, master orthopedic technician, state orthopedic technician, biomedical technician, speech therapist, state auxiliary midwife, state-registered nursing auxiliary, and pharmacy auxiliary.

Medical assistants are recognized by the Togolese Public Health Code in Article 159 as a paramedical profession that can be practiced without a state-recognized doctorate in medicine or dental surgery [12]. This includes senior health technicians specializing in general medicine, radiology and medical imaging, surgical instrumentation, odontostomatology, ophthalmology, and ENT. The survey focused on the sociodemographic characteristics, knowledge, perceptions, and attitudes of medical auxiliaries and assistants regarding telemedicine.

Knowledge refers to the objective concepts that respondents have acquired about telemedicine. Perceptions refer to respondents’ subjective views on this subject. Attitudes refer to respondents’ behaviors or practices in this area.

These parameters were used to develop our questionnaire, which included open short-answer questions, multiple-choice questions and closed questions.

We collected our data electronically and physically. We saved our form on Google Forms at the following URL: https://forms.gle/MSgdEvk2TSSC5squ8. We sent this URL by email and WhatsApp to medical auxiliaries and assistants, mainly via the various WhatsApp group platforms for medical auxiliaries and assistants that were available during the study period. These group platforms included those created by various medical auxiliary and assistant associations in Togo, as well as those of various hospitals in the country. We also printed physical forms for the providers to fill out directly on site in our presence.

All participants were informed of the study’s purpose and invited to participate if they consented. All information relating to the study was included in the Google form. Participants were also informed of their right to refuse participation or withdraw at any time during the data collection process. Respondents completed the forms on a strictly anonymous basis.

We performed data processing and statistical analysis using EPI INFO software, version 7.2.2.6. We performed chi-square or Fisher’s exact tests for quantitative variables and considered a difference to be statistically significant if the p-value was less than 0.05.

## Results

Our sample consisted of 315 medical auxiliaries and assistants, including 175 men and 140 women, for a sex ratio of 1.25. Most of the respondents were medical auxiliaries (76.2%), under the age of 35 (51.4%), and had a bachelor’s degree (78.7%). Most had been working for less than 10 years (58.1%) (Table I).

**Table I:** Distribution of medical auxiliaries and assistants by socio-demographic characteristics in Togo, 2021

	n	%
Age (in years)	315	
<25	38	12,1
[25-35 [	124	39,4
[35-45 [	114	36,2
[45-55 [	35	11,1
[55-65 [	2	0,6
[65-75]	2	0,6
Qualification	315	
Medical auxiliaries	240	76,2
Medical assistants	75	23,8
Professional experience (in years)	315	
< 5 (n=89)	89	28,3
[5-10] (n=94)	94	29,8
[10-15] (n=82)	82	26,0
>15 (n=50)	50	15,9
Study level	315	
Primary school leaving certificate	2	0,6
Junior High School Certificate	19	6,0
Baccalaureate	23	7,3
Bachelor’s Degree	248	78,7
Master’s Degree	23	7,3

Of the 240 medical auxiliaries, 114 were nurses (47.5%) and 82 were midwives (34.2%). Of the 75 medical assistants, 53.3% were general medicine assistants, also known as senior health technicians. Of those surveyed, 97.8% had been trained in Togo. Two hundred and twenty-one respondents (70.2%) worked in Lomé, while 94 (29.8%) worked in other cities across the country. Two hundred and eighty-four (90.2%) worked in public facilities, while the remaining 9.8% worked in private facilities.

Eighty-seven respondents rated the speed of their workplace internet connection as slow (27.6%), 100 poor (31.8%), 77 good (24.4%), and 8 very good (2.5%). Forty-three respondents (13.7%) reported that internet access was unavailable at their workplace.

Of the practitioners surveyed, 242 (76.8%) reported having heard of telemedicine at least once. The media was the most frequently cited source of information (Table II).

**Table II:** Distribution of means of information of medical auxiliaries and assistants who have already heard of telemedicine according to their cities of practice in Togo, 2021

	Lomé		Outside Lomé		Total	
	n	%	n	%	n	%
Colleague	35	14,5	17	7,0	52	21,5
Internet	64	26,4	25	10,3	89	36,8
Patients	6	2,5	0	0	6	2,5
Meeting	12	5,0	7	2,9	19	7,9
Medical journals	14	5,8	8	3,3	22	9,1
Theme evenings	3	1,2	1	0,4	4	1,7
Medias	72	29,7	33	13,6	105	43,4

Sixty-six respondents (21%) had heard of a telemedicine activity or project in Togo. Among the 315 respondents, 35.9% were unfamiliar with telemedicine procedures; 53% knew the teleconsultations; 18.1% telemedical monitoring; 15.2% telehealth assistance; 8.9% teleexpertise and 3.2% medical regulation. Knowledge of teleconsultation and expertise was more prevalent among respondents with a university education, with a statistically significant difference (p < 0.05).

Knowledge of telemonitoring and telehealth assistance, however, was significantly more prevalent (p < 0.05) among practitioners working in Lomé (Table III). Table III also shows that knowledge of telehealth assistance was more prevalent among respondents with a university education, with a statistically significant difference (p < 0.05).

**Table III:** Profile of medical auxiliaries and assistants familiar with the various telemedicine procedures in Togo, 2021

	Teleconsultation			Teleexpertise			Telemonitoring			Telehealth assistance		
	n	%	p	n	%	p	n	%	p	n	%	p
Age (in years)			0,482			0,154			0,170			0,176
< 35 (n=162)	89	54,9		18	11,11		34	21,0		29	17,9	
> 35 (n=153)	78	51,0		10	6,5		23	15,0		19	12,4	
Professional experience (in years)			0,567			0,077			0,908			0,634
< 5 (n=89)	49	55,0		9	10,1		16	18,0		16	18,0	
[5-10] (n=94)	50	53,2		9	10,0		17	18,1		13	13,8	
[10-15] (n=82)	43	52,4		9	11,0		16	19,5		11	13,4	
>15 (n=50)	25	50,0		1	2,0		8	16,0		8	16,0	
Place of work			0,006			0,878			0,004			0,030
Lomé (n=221)	106	48,0		20	9,0		49	22,2		40	18,0	
Outside Lomé (n=94)	61	64,9		8	8,5		8	8,5		8	8,5	
Study level			0,017			0,022			0,094			0,009
Non-university (n=44)	16	36,4		0	0		4	9,1		1	2,3	
University (n=271)	151	55,7		28	10,3		53	19,6		47	17,3	
Qualification			0,165			0,121			0,844			0,344
Auxiliary (n=240)	122	50,8		18	7,5		44	18,3		34	14,2	
Assistant (n=75)	45	60,0		10	13,3		13	17,3		14	18,7	

Of the respondents, 71 (22.5%) reported knowing ways to learn how to practice telemedicine. Of those, 41 (13%) cited videoconferencing, and 36 (11.4%) cited e-training. One hundred and fifty-three respondents (48.6%) knew it was important to obtain patient consent before collecting and sharing their information with colleagues or before any telemedicine procedure. Awareness of the importance of obtaining patient consent before a telemedicine procedure was more prevalent among medical assistants and among those practicing outside of Lomé (p < 0.05) (Table IV).

**Table IV:** Profile of respondents citing e-training and videoconferencing as a means of training for telemedicine practice and those aware of the need for patient consent prior to a telemedicine procedure in Togo, 2021

	e-learning			Videoconferencing			Patient consent		
	n	%	p	n	%	p	n	%	p
Age (in years)			0,378			0,977			0,877
< 35 (n=162)	21	13,0		21	13,0		78	48,2	
> 35(n=153)	15	9,8		20	13,1		75	49,0	
Professional experience (in years)			0,539			0,886			0,760
< 5 (n=89)	8	9,0		5	5,6		43	48,3	
[5-10] (n=94)	14	14,9		19	20,2		45	47,9	
[10-15] (n=82)	13	15,9		16	19,5		39	47,6	
>15 (n=50)	1	2,0		1	2,0		26	52,0	
Place of work			0,005			<0,001			<0,001
Lomé (n=221)	18	8,1		18	8,1		91	41,2	
Outside Lomé (n=94)	18	19,2		23	24,5		62	66,0	
Study level			0,599			0,002			0,656
Non-university (n=44)	4	9,1		0	0		20	45,5	
University (n=271)	32	11,8		41	15,1		133	49,1	
Qualification			0,154			0,039			0,045
Auxiliary (n=240)	24	10,0		26	10,8		109	45,4	
Assistant (n=75)	12	16,0		15	20,0		44	58,7	

The main benefits of telemedicine cited by the 315 respondents were benefits for patients (44.4%), combating medical desertification in rural areas (19.7%), and bringing patients closer to hospital facilities (19.1%). Respondents cited the following as the main barriers to telemedicine: economic barriers (64.8%), organizational barriers (54.3%), lack of motivation (37.1%), lack of credibility with patients (36.8%), and incomplete examinations (35.6%).

Two hundred and fifty-nine respondents (82.2%) considered telemedicine useful in their daily practice. Telemedicine was perceived as a necessity by 142 respondents (45.1%), as the hope and future of medicine by 133 respondents (42.2%), and as a matter for specialist doctors by 26 respondents (8.3%). A total of 285 respondents (90.5%) expressed interest in telemedicine training. The respondents outside of Lomé perceived telemedicine as a necessity more often than those in Lomé. Those working in Lomé and medical auxiliaries perceived it as the hope and future of medicine more often. There was a statistically significant difference between the two groups (p<0.05) (Table V). Table V also shows that practitioners over 35 years of age, those with more than 15 years of professional experience, and those practicing outside Lomé expressed a significantly greater desire for training (p<0.05).

**Table V:** Profile of respondents considering telemedicine as a necessity, a hope and a future of medicine, and those willing to train in the practice of telemedicine in Togo, 2021

	Telemedicine as a necessity			Telemedicine as hope and the future of medicine			Desire for telemedicine training		
	n	%	p	n	%	p	n	%	p
Age (in years)			0,361			0,050			0,032
< 35 (n=162)	69	42,6		77	47,5		141	87,0	
> 35(n=153)	73	47,7		56	36,6		14	94,1	
Professional experience (in years)			0,326			0,367			0,046
< 5 (n=89)	36	40,5		40	44,9		78	87,6	
[5-10] (n=94)	43	45,7		42	44,7		82	87,2	
[10-15] (n=82)	39	47,6		31	37,8		77	94,0	
>15 (n=50)	24	48,0		20	40,0		48	96,0	
Place of work			0,004			0,016			0,038
Lomé (n=221)	88	39,8		103	46,6		195	88,2	
Outside Lomé (n=94)	54	57,5		30	31,9		90	96,0	
Study level			1,0			0,849			1,000
Non-university (n=44)	20	45,5		18	41,0		40	90,9	
University (n=271)	122	45,0		115	42,4		245	90,4	
Qualification			0,1			0,0			0,3
Auxiliary (n=240)	102	42,5		109	45,4		215	89,6	
Assistant (n=75)	40	53,3		24	32,0		70	93,3	

According to the surveys, general medicine was deemed the field that would benefit most from the development of telemedicine, at 74.3%. The next most likely fields were medical imaging (41%), dermatology (38.4%), pediatrics (29.2%), surgery (27%), cardiology (26.7%), psychiatry (26.7%), and gynecology and obstetrics (6.4%). Of the respondents, 27.3% reported practicing telemonitoring; 24.1%, medical teleassistance; 9.2%, teleexpertise; and 3.5%, medical regulation. One hundred sixty-nine respondents (53.7%) reported never having performed any telemedicine activities. Medical telemonitoring was significantly more common among younger respondents (under 35) and those with less than five years of professional experience (Table VI).

**Table VI:** Profile of medical auxiliaries and assistants who have already practiced telemonitoring and telehealth assistance in Togo in 2021

	Practice of medical telemonitoring			Practice of telehealth assistance		
	n	%	p	n	%	p
Age (in years)			0,045			0,592
< 35 (n=162)	52	32,1		41	25,3	
> 35(n=153)	34	22,2		35	22,9	
Professional experience (in years)			0,043			0,481
< 5 (n=89)	30	33,7		19	21,4	
[5-10] (n=94)	26	27,7		24	25,5	
[10-15] (n=82)	21	25,6		19	23,2	
>15 (n=50)	9	18,0		14	28,0	
Place of work			0,328			0,314
Lomé (n=221)	57	25,8		50	22,6	
Outside Lomé (n=94)	29	30,9		26	27,7	
Study level			0,3			0,6
Non-university (n=44)	9	20,5		9	20,5	
University (n=271)	77	28,4		67	24,7	
Qualification			0,1			0,6
Auxiliary (n=240)	71	29,6		56	23,3	
Assistant (n=75)	15	20,0		20	26,7	

Of the respondents, 155 (49.2%) and 88 (27.9%) had never used remote consultation or prescription services, respectively. Additionally, 87 (27.6%) and 105 (33.3%) had never used virtual channels to receive paraclinical results from patients or colleagues, respectively. Respondents primarily used the WhatsApp application for remote monitoring, medical prescriptions, and receiving results from patients and colleagues (Table VII). Of the respondents, 296 (94.0%) said they had never used connected devices to take patients’ vital signs, while 19 (6%) said they had used a connected device.

**Table VII:** Distribution of monitoring, remote prescribing and virtual channels for receiving paraclinical results used by medical auxiliaries and assistants

	Remote monitoring		Remote prescribing		Receiving results from patients		Receiving results from colleagues	
	n	%	n	%	n	%	n	%
WhatsApp	157	49,8	222	70,5	222	70,5	206	65,4
Facebook	4	1,3	5	1,6	1	0,3	1	0,3
Other social networks	18	5,7	15	4,8	15	4,8	3	1,0

The preferred places for practicing telemedicine among the 315 medical auxiliaries and assistants were public facilities (65.4%), homes (50.5%), and private practices (43.8%).

## Discussion

Our study focuses on non-physician healthcare professionals, including medical auxiliaries and assistants, whose role in patient care is essential in any healthcare setting. Most of the medical auxiliaries in our study (81.8%) were nurses and midwives, the most numerous professions and the ones most closely involved with doctors in hospital care.

Due to a shortage of doctors, particularly in rural areas, Togo’s health authorities have trained intermediate health professionals to bridge the gap between medical auxiliaries and general practitioners. These professionals perform general practitioner duties by delegation in health centers that lack a doctor. Medical assistants specializing in general medicine are known as senior health technicians in accordance with the Togolese Health Code [12]. They are the most operational and therefore the largest group in our sample (53.3%).

The sex ratio of respondents, 1.25, differs from the ratio (0.87) found by Ayatollahi *et al.* in their study on clinicians’ knowledge and perceptions of telemedicine in Iran [4]. Our findings align with the most recent activity report from the Togolese Ministry of Health and Public Hygiene, which revealed a sex ratio of 1.30 among public sector healthcare personnel [14]. This reflects the gender representation of our sample.

Unfortunately, around a quarter of the practitioners surveyed in our study had never heard of telemedicine in 2021, despite the fact that the usefulness of telemedicine as a means of implementing social distancing was highlighted by the Covid-19 pandemic during this period. This result may reflect an insufficient communication strategy by the Togolese health authorities regarding telemedicine during the pandemic that emerged in 2019.

In our study, the media was the most frequently cited source of information on telemedicine (43%), followed by the internet (36.8%) and colleagues (21.5%). Shittu *et al.* found similar results in their Nigerian study: the media were the most frequently cited source of information on telemedicine, followed by the internet and colleagues [19]. Gaggioli *et al.,* in their 2005 survey of doctors in Milan, Italy, found that medical journals were the most frequently cited source of information on telemedicine (46%), followed by colleagues (41%), scientific conferences (26%), television (19%), and the internet (10%) [10]. Messon’s 2018 survey of general practitioners in Gironde, France, found that medical journals ranked first (66%), followed by the internet (52.1%), colleagues (48.3%), television (42.3%), and patients (6%) [13]. The fact that medical journals ranked first in studies conducted in Western countries (Italy and France) but not in African studies (ours and the one conducted in Nigeria) can be explained by the greater accessibility of medical journals for doctors in these countries.

Telemedicine can be divided into five main categories: teleconsultation, teleexpertise, telemonitoring, telemedical assistance, and medical regulation. However, our study found that approximately 36% of respondents were unaware of any form of telemedicine. Fifty-three percent of respondents were familiar with teleconsultation, which allows a medical professional to consult with a patient remotely. Teleexpertise, which was unknown to about 81% of respondents, enables a medical professional to seek the advice of one or more specialists remotely based on the patient’s medical data. The patient does not attend the session. Telemedical monitoring enables a healthcare professional to interpret data necessary for a patient’s medical follow-up remotely and, if necessary, make decisions regarding their care. Data recording and transmission may be automated or carried out by the patient or a healthcare professional. Only 18.1% of respondents were familiar with it, even though it often involves medical auxiliaries, such as nurses and midwives. Medical telecare, known to 15.2% of respondents, allows a medical professional to assist another healthcare professional remotely during a medical procedure. Finally, medical regulation, the least known procedure among respondents at 3.1%, is a medical procedure performed over the phone or by any other means of telecommunication by a medical regulator. The purpose of medical regulation is to determine and initiate the appropriate medical response to each situation as quickly as possible. Practitioners working in Lomé were more familiar with medical teleassistance and telemonitoring. This can be explained by the fact that large cities, such as Lomé (the capital of Togo), offer more opportunities to learn about medical advances than rural areas, where access to information is often limited. In a 2019 survey of general practitioners working in multi-professional health centers in Burgundy, France, Berou found that 82.9% of doctors were familiar with teleconsultation, 80% with teleexpertise, 47.7% with medical telemonitoring, and 20% with medical teleassistance [6]. Berou’s results are more favorable than ours, which is logical given that his study concerned doctors practicing in a developed country.

The practice of telemedicine must adhere to ethical requirements, with patient consent being a key consideration [7,20]. However, only 48.6% of practitioners surveyed were aware of the importance of obtaining consent before a telemedicine procedure.

Our results show that economic factors were cited more frequently in Togo than in Gironde as barriers to telemedicine. Messon found that 55% of doctors cited economic factors, such as financing, in his study [13], compared to 65% in ours. This finding reflects the reality in developing countries, where economic and organizational barriers remain a major obstacle to the development of telemedicine. Indeed, most telemedicine initiatives in sub-Saharan Africa do not progress beyond the pilot stage. These barriers are primarily due to the high cost of equipment, limited access to high-speed internet, low healthcare facility budgets, and the absence of a viable economic model. Organizational barriers to telemedicine in Africa include a lack of standardized protocols, a shortage of trained personnel, difficulties integrating telemedicine into clinical routines, and flaws in data management. In addition to economic and organizational difficulties, 37.1% of respondents identified a lack of motivation (often associated with resistance to change) as a barrier. This result may be due to a lack of initiative or fear of change in our societies, particularly in Africa.

In our study, 82.2% of respondents found telemedicine to be useful in everyday medical practice. Shittu *et al.* found that 78.1% of Nigerian healthcare workers were willing to adopt telemedicine for healthcare delivery if it were available [19]. Similarly, in a 2013 study in Iran, Ayatollahi *et al.* reported that 74.8% of clinicians agreed with the need for telemedicine technologies [4]. However, Messon found that only 46% of doctors in Gironde considered telemedicine useful in everyday medical practice [13]. The more positive perception of telemedicine in developing countries, particularly in Togo, is encouraging and represents an opportunity for growth of telemedicine in this country. Respondents practicing outside of Lomé perceived telemedicine as more necessary than those in Lomé. It can be explained by the technical and professional isolation of health personnel working in rural areas, especially in developing countries. These professionals are more likely to need advice from colleagues or specialists practicing in the capital or large cities. In our study, WhatsApp and Facebook were the primary means of remote telemedicine used by the respondents. Similarly, Albarak *et al.* found that, in 2019, approximately 72% of doctors in Riyadh, Saudi Arabia, interacted with their patients via email or social media [3]. It is not surprising that social media platforms like WhatsApp and Facebook are among the main means of practicing telemedicine. Since their inception (WhatsApp in 2009 and Facebook in 2004), social media have grown rapidly and become indispensable in all areas, especially communication. However, some platforms, such as WhatsApp and especially Facebook, are not suitable for secure telemedicine. Due to the security risks they pose, particularly with regard to confidentiality, they should be avoided for transmitting patient data during telemedicine activities. A medicolegal requirement for both healthcare providers and patients when using telemedicine is to ensure the security of transmitted data. It is important to use a secure health messaging service to exchange information and avoid personal email services.

According to our findings, 46.2% of respondents reported having undergone a telemedicine procedure. A significant difference in telemonitoring was observed between younger respondents (under 35) and those with less than five years of professional experience. These results suggest that telemedicine practices by medical auxiliaries and assistants will increase in Togo in the coming years. Provisions must be made to train this staff in proper telemedicine practices. In our study, the vast majority (90.5%) of respondents strongly requested continuing education on telemedicine, which is essential for any healthcare professional wishing to integrate it into their daily practice [9]. In Western countries, connected devices are increasingly being used for patient monitoring, particularly remotely. In France, for instance, Le Guillou *et al.* [11] found that one-third of healthcare workers used connected devices to monitor patients. This is not yet the case in Togo. Only 6% of medical auxiliaries and assistants reported ever using a connected device to remotely monitor or take a patient’s vital signs.

Finally, most respondents considered their workplace internet connection speed to be unsatisfactory. The same was true for imaging physicians in French-speaking sub-Saharan Africa in a previous study [1]. These results demonstrate that equipping hospitals with high-quality internet connections with sufficient bandwidth remains a challenge for health authorities in our countries.

Using two data collection methods (electronic and paper forms) is a potential limitation of our study. Respondents who completed paper forms directly may have introduced bias into their responses. Similarly, respondents who completed electronic forms may have consulted documents before answering, which could introduce other bias into the data.

## Conclusion

This cross-sectional study of non-physician healthcare workers in Togo revealed a relatively low level of knowledge about telemedicine among respondents. However, they had a positive perception of telemedicine, considering it useful in their daily medical practice. The most common activities performed by these medical auxiliaries and assistants were telemonitoring and medical teleassistance. Virtual channels with low levels of data security, such as WhatsApp, were the most commonly used. To ensure the efficient and secure use of telemedicine for patients’ benefit, it is desirable to provide continuing medical education on telemedicine to improve the knowledge level of medical auxiliaries and assistants in Togo.

## Ethical approval and consent to participate

Approval was obtained from the thesis committee of the Faculty of Health Sciences at the University of Lomé. This study did not involve the use of animals and was conducted in accordance with the Declaration of Helsinki. This manuscript has not been submitted to any other journal or website, in part or in full, for consideration. It is submitted solely to this journal. All medical auxiliaries included in this study gave their informed consent to participate in this research.

## Availability of data and materials

The data used and/or analyzed during this study are available from the corresponding author upon reasonable request.

## Funding

This research did not receive any specific funding from public, commercial, or nonprofit agencies.

## Authors’ contributions

Kokou ADAMBOUNO: Lead author, initiator of the work, data collection, methodology, critical revision, and final approval of the published version.

Rarba Baniki MOROUMA-TISSOGANA: Contributed to data collection and manuscript writing. Final approval of the version to be published.

Akoeté Beleave KOUVIDJIN: Contributed to the manuscript and approved the final version.

## Conflict of interest

The authors declare no conflicts of interest.

## Appendix 1: Survey form

**A - Sociodemographic characteristics of medical assistants and aides**

1 - Gender: Male /___________/ Female /___________/

2 - Age

< 25 years /___________/ 26-35 years /___________/ 36-45 years /___________/ 46-55 years /___________/ 56-65 years/___________/ > 65 years /___________/

3 - Qualification of medical auxiliaries and assistants:

Registered nurse/___________/ Midwife/___________/ Physical therapist /___________/ Speech therapist /___________/ Senior healthcare technician (general medicine) /___________/ Medical assistant/Healthcare administrative manager /___________/ Anesthesia and intensive care assistant /___________/ Medical assistant in anesthesia and intensive care /___________/ Medical assistant in radiology and medical imaging /___________/ Medical assistant in surgical instrumentation in operating rooms /___________/ Medical assistant in odontostomatology /___________/ Medical assistant in mental health /___________/ Other: ………………………..

4 - Level of education: Primary school leaving certificate /___________/ Junior High School Certificate /___________/ Baccalaureate/___________/ Bachelor’s degree /___________/ Master’s degree /___________/

5 - Professional experience (years): 0-4 /___________/ 5-9 /___________/ 10-15 /___________/ > 15 /___________/

6 - Country of training: Togo /___________/ Other: ………………………..

7 - Place of work: Lomé /___________/ Outside Lomé: ………………………..

8 - Administrative structure for practice:

CHU /___________/ CHR /___________/ CHP /___________/ CMS /___________/ Private clinic /___________/ Other………………………..

9 - What is the speed of the internet connection at your place of work?

Very fast /___________/ Good /___________/ Poor /___________/ No internet connection /___________/

**B - Knowledge of medical auxiliaries and assistants about telemedicine in Togo**

10 - Have you ever heard of telemedicine? No /___________/ Yes /___________/

11 - If so, how?

Media /___________/ Medical journal /___________/ Themed evening /___________/ Meeting /___________/ With a colleague /___________/ Patients /___________/ Internet /___________/ Other: ………………………..

12 - Which of the following telemedicine procedures are you familiar with?

Teleconsultation /___________/ Teleexpertise /___________/ Telemonitoring /___________/ Telehealth assistance /___________/

Medical regulation /___________/ None /___________/

13a - What solutions/applications/means of exercising remotely are you familiar with?

…………………………………………………………………………………………………….

…………………………………………………………………………………………………….

13b - Which ones do you use?

…………………………………………………………………………………………………….

…………………………………………………………………………………………………….

14a - Are you aware of any training opportunities for learning how to practice telemedicine? No /___________/ Yes /___________/

14b - If yes: which one(s)?

E-learning /___________/ Videoconferencing /___________/ Inter-university degree /___________/ Telemedicine Day /___________/

Other: ………………………..

15 - Have you ever heard of any past or current telemedicine activities or projects in Togo? No /___________/ Yes /___________/

16 - What do you think will be the main benefit of using telemedicine in your practice?

Collaboration with a hospital facility (contact with specialists) /___________/

Benefits for patients (time savings/less travel) /___________/

Helping to combat the decline in medical services in rural areas /___________/ Distance learning /___________/

Telemonitoring /___________/ Other …………………………………..

17 - What do you think are the main obstacles to using telemedicine?

Organizational barriers (restructuring to implement such a technique) /___ /

Economic barriers (financing development, remuneration still unclear) /___________/ Lack of motivation /___________/

Lack of time (fear of wasting time with such a practice) /___________/ Lack of credibility with patients /___________/

Incomplete examination /___________/ Forensic aspect /___________/ None /___________/ Other………………………..

**C - Perceptions of medical auxiliaries and assistants regarding telemedicine in Togo**

18 - Do you think telemedicine is useful in your daily practice?

No /___________/ Yes /___________/

19 - For you, telemedicine is:

The future of medicine /___________/ Science fiction /___________/ A necessity /___________/ A matter for specialists /___________/ Hope /___________/ Not relevant /___________/

20 - In which areas would it be interesting to develop telemedicine services?

General medicine /___ / Medical imaging /___ / Dermatology /___ / Pediatrics /___ / Cardiology /___ / Surgery /___________/ Psychiatry /___________/ Other………………………..

21 - Would you like to learn this practice? No /___ / Yes /___ /

**D - Attitudes of medical assistants and aides toward telemedicine in Togo**

22 - Which of the following telemedicine procedures do you perform?

Telemedicine /___ / Medical telemonitoring /___ / Medical teleassistance /___ / Medical regulation /___ / None /___________/

23 - Have you ever monitored a patient through one of these virtual channels?

WhatsApp /___________/ Facebook /___________/ Other social networks /___________/ Never /___________/ Other………………………..

24 -Have you ever prescribed medication to a patient through one or more of these virtual channels?

WhatsApp /___________/ Facebook /___________/ Other social networks /___________/ Never /___________/ Other………………………..

25 - Have you ever received a paraclinical result from a patient via one or more of these virtual channels?

WhatsApp /___________/ Facebook /___________/ Other social networks /___________/ Never /___________/ Other………………………..

26 - Have you ever received a paraclinical result from a colleague via one or more of these virtual channels?

WhatsApp /___________/ Facebook /___________/ AOther social networks /___________/ Never /___________/ Other………………………..

27 - Have you ever taken a patient’s vital signs using one or more of these connected devices?

Smartwatch /___ / Smart bracelet /___ / Connected scale /___ / Jamais /___ /

28 - Where would you be willing to practice telemedicine?

Your home /___________/ Private practice /___________/ Public institution /___________/ None /___________/ Other………………………..

29 - Has the patient’s consent been obtained for the collection and sharing of their information with other colleagues?

No /___________/ Yes /___________/
